# Prediction Model of Osteonecrosis of the Femoral Head After Femoral Neck Fracture: Machine Learning–Based Development and Validation Study

**DOI:** 10.2196/30079

**Published:** 2021-11-19

**Authors:** Huan Wang, Wei Wu, Chunxia Han, Jiaqi Zheng, Xinyu Cai, Shimin Chang, Junlong Shi, Nan Xu, Zisheng Ai

**Affiliations:** 1 Department of Medical Statistics Tongji University School of Medicine Shanghai China; 2 Department of Spinal Surgery Shanghai East Hospital Shanghai China; 3 Department of Orthopedics Shanghai Tenth People's Hospital Shanghai China; 4 Department of Orthopedic Surgery Yangpu Hospital, Tongji University School of Medicine Shanghai China; 5 Medical Record Department Shanghai Ninth People’s Hospital Shanghai China; 6 Department of Radiology Shanghai East Hospital Shanghai China

**Keywords:** femoral neck fracture, osteonecrosis of the femoral head, machine learning, interpretability

## Abstract

**Background:**

The absolute number of femoral neck fractures (FNFs) is increasing; however, the prediction of traumatic femoral head necrosis remains difficult. Machine learning algorithms have the potential to be superior to traditional prediction methods for the prediction of traumatic femoral head necrosis.

**Objective:**

The aim of this study is to use machine learning to construct a model for the analysis of risk factors and prediction of osteonecrosis of the femoral head (ONFH) in patients with FNF after internal fixation.

**Methods:**

We retrospectively collected preoperative, intraoperative, and postoperative clinical data of patients with FNF in 4 hospitals in Shanghai and followed up the patients for more than 2.5 years. A total of 259 patients with 43 variables were included in the study. The data were randomly divided into a training set (181/259, 69.8%) and a validation set (78/259, 30.1%). External data (n=376) were obtained from a retrospective cohort study of patients with FNF in 3 other hospitals. Least absolute shrinkage and selection operator regression and the support vector machine algorithm were used for variable selection. Logistic regression, random forest, support vector machine, and eXtreme Gradient Boosting (XGBoost) were used to develop the model on the training set. The validation set was used to tune the model hyperparameters to determine the final prediction model, and the external data were used to compare and evaluate the model performance. We compared the accuracy, discrimination, and calibration of the models to identify the best machine learning algorithm for predicting ONFH. Shapley additive explanations and local interpretable model-agnostic explanations were used to determine the interpretability of the black box model.

**Results:**

A total of 11 variables were selected for the models. The XGBoost model performed best on the validation set and external data. The accuracy, sensitivity, and area under the receiver operating characteristic curve of the model on the validation set were 0.987, 0.929, and 0.992, respectively. The accuracy, sensitivity, specificity, and area under the receiver operating characteristic curve of the model on the external data were 0.907, 0.807, 0.935, and 0.933, respectively, and the log-loss was 0.279. The calibration curve demonstrated good agreement between the predicted probability and actual risk. The interpretability of the features and individual predictions were realized using the Shapley additive explanations and local interpretable model-agnostic explanations algorithms. In addition, the XGBoost model was translated into a self-made web-based risk calculator to estimate an individual’s probability of ONFH.

**Conclusions:**

Machine learning performs well in predicting ONFH after internal fixation of FNF. The 6-variable XGBoost model predicted the risk of ONFH well and had good generalization ability on the external data, which can be used for the clinical prediction of ONFH after internal fixation of FNF.

## Introduction

### Background

The incidence of hip fractures is changing worldwide. In most Western and Northern European countries, the incidence is decreasing, as well as in Singapore [1-5]. The incidence in China and America is stabilizing [6,7], whereas that in Germany, Japan, and Korea is still increasing [8-10]. Although the age-adjusted incidence of hip fractures is declining or stabilizing in some countries, the absolute number of hip fractures and the costs of associated medical care are still increasing. Femoral neck fractures (FNFs) account for approximately 48.22% to 52.26% of hip fractures [9,11,12], and 23% of young patients have osteonecrosis of the femoral head (ONFH) after internal fixation [13]. Early stages of ONFH allow hip joint preservation surgery, such as free fibula transplantation and osteotomy, before collapse of the femoral head occurs [14]. A previous study demonstrated that the short-term and medium-term success rates of early hip-preserving treatment of ONFH were between 55% and 87% [15,16].

Published papers regarding ONFH prediction are primarily based on changes in the blood circulation of the femoral head by radiological investigations, such as single-photon emission computed tomography [17]/single-photon emission computed tomography-computed tomography [18], positron emission tomography [19]/positron emission tomography-computed tomography [20], magnetic resonance imaging [21]/dynamic contrast-enhanced-magnetic resonance imaging [22], and digital subtraction angiography [23]. The sample sizes of most studies were not large enough, and their prediction results were not confirmed in subsequent prospective studies. Cui et al [24] first applied machine learning to predict small samples of ONFH. However, the accuracy, sensitivity, and area under the receiver operating characteristic curve (AUC) of the model based on naive Bayes were all lower than 80%. Zheng et al [25] and Zhu et al [26] developed a nomogram for the risk assessment of femoral head necrosis based on a traditional regression analysis. The AUCs of the validation cohort were 0.94 and 0.95. However, these studies lacked external validation.

Prediction research using machine learning involves learning models from sample data and making predictions and decisions on the new data. The support vector machine (SVM) algorithm exhibits good prediction performance and generalization ability when dealing with small sample binary classification problems [27]. Random forest (RF) and eXtreme Gradient Boosting (XGBoost) are ensemble learning algorithms that establish multiple models using the data and then integrate the modeling results from all models. Currently, they are the most popular models in the industry.

In machine learning, the black box describes models that cannot be understood by examining their parameters (eg, neural network and XGBoost) [28]. Interpretability is defined as the ability to explain or provide meaning in understandable terms to a human. The pursuit of interpretability of the black box model helps to improve users’ trust in the machine learning model and provides support for human decision-making. Arrieta et al [29] summarized and distinguished between transparent models and those that can be interpreted by post hoc explainability techniques. Transparent models convey some degree of interpretability by themselves, such as logistic regression (LR) and decision trees. Post hoc explainability techniques are model-agnostic methods, including local explanations, explanations by simplification, and feature relevance explanation techniques. Further exploration based on these methods could help overcome the difficulties related to explainability and make the machine learning models more persuasive and dependable.

### Objectives

This study aims to explore and compare the application value of different machine learning algorithms for the prediction of ONFH after internal fixation of FNF and to develop a prediction model of ONFH based on a machine learning algorithm. In this study, the development and validation of the prediction model was guided by the Prediction Model Risk of Bias Assessment Tool (PROBAST) [30] and adhered to the Transparent Reporting of a Multivariable Prediction Model for Individual Prognosis or Diagnosis (TRIPOD) [31] statement for reporting. To provide appropriate models to the clinic, the design, implementation, and report also considered the 20 key issues raised by Vollmer et al [32] regarding transparency, reproducibility, ethics, and effectiveness of the study.

## Methods

### Study Population

This multicenter retrospective follow-up study was performed at least 30 months after follow-up in patients undergoing internal fixation of FNFs. The study population comprised patients with FNF with internal fixation who were discharged from Shanghai Ninth People’s Hospital, Dongfang Hospital, and Yangpu District Central Hospital from January 1, 2015, to May 1, 2018, and the Tenth People’s Hospital from January 1, 2017, to May 1, 2018. By searching the inpatient electronic medical record system, medical imaging information system, laboratory information system, manual reading of cases, and follow-up, we collected 47 clinical features from 316 patients with FNF. After excluding patients who were lost to follow-up or who died, 259 patients with FNF and associated 43 variables were included in this study. The external data (n=376) were obtained from our previous retrospective cohort study [25], which collected data from a cohort of patients with FNFs from May 2013 to January 2017 at Shanghai Sixth Hospital, Tenth Hospital, and Tongji Hospital. Two patients aged >75 years were excluded.

The inclusion criteria were as follows: (1) patients with FNF treated with internal fixation aged between 18 and 75 years, (2) patients with FNF with complete baseline data, (3) follow-up time ≥30 months, (4) The American Society of Anesthesiologists anesthesia risk of approximately grade I-III, and (5) no moderate or severe pain or limitation of movement of the injured hip side that occurred before the fracture. Exclusion criteria included the following: (1) patients with FNF with pathological fractures or old fractures admitted for >2 weeks after injury; (2) patients with failed internal fixation operation; (3) patients with a history of malignant tumors, nontraumatic fractures, ipsilateral lower limb, or other fractures; (4) patients with a history of long-term diving, alcohol abuse, and fluoroquinolone, antiplatelet drug, or hormone use; (5) patients with multiple fractures at the same site, injuries on the opposite side, or fracture of both lower limbs in the past 6 months; (6) patients who experienced acute myocardial infarction, cerebrovascular accident, severe trauma, or major operation within half a year; (7) patients with vascular transplantation or free fibula transplantation during internal fixation; and (8) patients with poor compliance. The diagnosis of ONFH was based on the updated version of the Association Research Circulation Osseous grading system [14], which was displayed and approved at the Association Research Circulation Osseous conference in Dalian, China, in 2019, and was used at the same time as the ONFH Chinese grading system developed in China in 2015 [33].

### Ethics Statement

The protocol for this research project was approved by the Medical and Life Science Ethics Committee of Tongji University (2019tjdx285; date June 18, 2019). Given the retrospective nature of this study, the requirement for informed consent was waived.

### Independent Variables (Features)

A total of 47 clinical features were collected, and features with missing values >20% were excluded. Overall, 43 candidate variables were included in the following categories: (1) demographic information: age, sex, smoking, drinking, and age-adjusted Charlson Comorbidity Index [34]; (2) fracture related: injury cause [35], injured side, fracture position, impaction, preoperative displacement, vertical axis of the neck angle [36], and Garden classification [37]; (3) preoperative biochemical characteristics: total protein, albumin, albumin/globulin, total bilirubin, alanine aminotransferase, aspartate aminotransferase, creatinine, uric acid, and urea nitrogen; (4) preoperative routine blood parameters: red blood cells, hemoglobin, white blood cells, platelets, and hematocrit; (5) preoperative coagulation parameters: prothrombin time, fibrinogen, activated partial thromboplastin time, and international normalized ratio; (6) surgery-related parameters: American Society of Anesthesiologists grade, time to surgery, type of anesthesia, surgical treatment, and surgical method; (7) postoperative related characteristics: reduction quality [38], Lowell curve, Gotfried reduction [39], and femoral neck shortening [40]; and (8) follow-up information: interval to part weightbearing, interval to weightbearing, implant removal, and visual analog scale (VAS) score [41]. The values and definitions of the variables are presented in Multimedia Appendix 1.

### Data Preprocessing

Outlier detection was performed on the raw data. Each source of outlier was checked by looking through the medical history twice, so that we knew whether the value of the outlier was true. The errors caused by incorrect manual collection were rectified. In this study, the proportion of missing variables was <5% and was substituted with the mean value. The original data, such as blood biochemical indices, were continuous variables, which were converted into low, normal, and high categorical variables according to clinical significance. According to the modeling requirements, categorical variables were transformed into dummy variables. Standardization of continuous variables was not a necessary step in preprocessing. Although there were few continuous variables included in this study, we compared the effects of standardization with nonstandardization during the modeling process. Finally, the processed data were randomly divided into a training set and validation set at a ratio of 7:3.

### Development of Prediction Models

#### Data Balance

The ratio of ONFH to non-ONFH was 1:5, which is unbalanced. When unbalanced data are used to fit the model, the classification interface will be biased toward the minority, resulting in low sensitivity and high specificity [42]. To address this issue, we used the synthetic minority oversampling technique (SMOTE) algorithm to balance the training set. The steps of the SMOTE algorithm [43] are as follows: (1) for each sample X in the minority sample set, use the Euclidean distance as the standard to calculate the distance from all samples in the minority sample set to obtain its K nearest neighbors; and (2) set the sampling ratio according to the sample imbalance ratio and determine the sampling magnification N. For each minority sample X, randomly select several samples from its K nearest neighbors, assuming that the selected nearest neighbor is xn; (3) for each randomly selected neighbor xn, combine the original sample to construct a new sample according to the following formula: 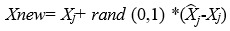 where X*_j_* is the sample in a few classes used to synthesize new samples, 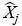 is the nearest neighbor. Although the features of adjacent points in the *feature space* are similar, the newly synthesized sample set will not affect the spatial boundary of the original minority samples.

#### Variable Selection

A large amount of collected clinical data will inevitably contain redundant features and noise data, which will lead to overfitting in modeling and cannot be effectively classified. Variable selection is a process that can remove irrelevant and redundant features and reduce the impact of noise data on classifier performance to a certain extent [44]. We used a combination of least absolute shrinkage and selection operator (LASSO) regression and the SVM algorithm for variable selection.

#### Modeling and Parameter Adjustment

Four classification algorithms, LR, RF, SVM, and XGBoost, were used to establish the models. The parameter learning curve, grid search, and cross-validation methods were used to adjust the parameters of the model, and the best parameter combination was determined by checking the accuracy, sensitivity, and AUC of the model on the validation set. The parameter learning curve is a curve with different parameter values as the abscissa, and the model score under different parameter values as the ordinate. We can see that the change in trend of the model evaluation index under different parameter values, initially obtain a small parameter search interval, or select the value of the best point of the model performance as the optimal parameter value. Grid search refers to the selection of all candidate parameters through loop traversal. The system tries every possibility, and the best-performing parameter is the final result, which is the process of training and comparison. Cross-validation refers to randomly dividing the data set into K parts without replacement, k-1 parts are used to train the model, and the remaining part is used for performance evaluation. This process was repeated K times to obtain k models and performance evaluation results.

#### Evaluation of Model Fitting Effect

The aim of parameter tuning is to minimize the generalization error of the model. The generalization error was used to measure the accuracy of the model with unknown data in machine learning. A model that is too simple or too complex will cause high generalization errors. If the model is too complex, it will overfit; if the model is too simple, it will underfit. By comparing the sample learning curves of the training with validation sets, we can observe the fitting effect of the model. The sample learning curve is drawn with the number of different training samples as the abscissa, and the accuracy of the training or validation sets under the number of samples as the ordinate. When the errors of the training and validation sets converge but the accuracy is low, it indicates a high bias. When the deviation of the upper left corner of the curve is very large and the accuracy of the training and validation sets is very low, the model is underfitted. When the errors of the training and validation sets are large, there is high variance; the variance in the upper right corner of the curve is high, the accuracy of the training and validation sets are too different, and the model is overfitted. If one of the biases and variances is large, this indicates that the generalization error is large.

#### Model Evaluation and Comparison

Confusion matrix indicates the count of the true outcome and prediction under different labels (ONFH or non-ONFH). A series of indicators can be calculated using the confusion matrix. The accuracy of the model is a key indicator for measuring the quality of the model. According to PROBAST [30], reviewers should evaluate the model performance, including discrimination and calibration ability. The receiver operating characteristic (ROC) curve reflects the dynamic relationship between the false-positive rate (FPR) and sensitivity (also known as recall). Different FPRs and sensitivities were obtained by classifying different predicted values corresponding to each point on the ROC curve. The area under the ROC curve is known as AUC, which is also considered as the possibility of the fact that *sensitivity is larger than FPR*. In addition, the precision-recall (PR) curve reflects the dynamic relationship between the precision and sensitivity. The area under the PR curve equals the average precision (AP), which is the AP at all thresholds. A larger AP usually indicates better discrimination ability, so does AUC. In particular, PR curves are available for unbalanced data [45]. By evaluating the fraction of true positives among positive predictions and actual positives (real ONFH patients), we illustrated the discrimination ability more properly and specifically. In this study, sensitivity, specificity, F1 score, ROC curve, and PR curve were used to evaluate discrimination ability.

Calibration includes log-loss and the calibration curve. Log-loss is the negative logarithm of the probability of the real probability for a given probability classifier under the condition of prediction probability. A smaller value of the log-likelihood function indicates a more accurate prediction. In this study, all samples were reordered according to the predicted probability and divided into 10 equal groups. The calibration curve showed the distance between the predicted probabilities and the true incidence of ONFH in each group. A curve closer to the ideal line (y=x) shows a better calibration ability of the model.

#### Model Interpretability

The black box model is explained through both global and local explanations. Shapley additive explanations (SHAP) is based on the theoretically optimal Shapley values [28]. The Shapley value explains the prediction of instance x by computing the contribution of each feature to the prediction. For each prediction sample, the model produces a prediction value, and the sum or average of the Shapley absolute value of each feature of all individuals is the overall feature importance. Features with large absolute Shapley values are very important; therefore, the importance of features can be ranked from a global perspective according to the absolute value of Shapley. The local interpretable model-agnostic explanations (LIME) algorithm obtains the probability value of each category by selecting specific samples in the data set and explains the reason for the distribution probability. LIME decomposes the sample space into parts and attempts to use simple models (such as linear models) that are easy to explain to fit complex models that are not easy to explain. LIME focuses on training local surrogate models to explain individual predictions [28].

### Statistical Analysis

Qualitative variables are expressed as ratios or constituent ratios. The Kolmogorov-Smirnov test was used to test the normality of the quantitative variables. Variables that fit the normal distribution were expressed as the mean (SD), and variables that did not fit the normal distribution were expressed as the median (25th percentile [P_25_] and 75th percentile [P_75_]). The Kendall correlation coefficient and Spearman correlation coefficient were used to describe the correlation between the qualitative and quantitative variables, respectively. A coefficient greater than 0.6 indicates that there is a correlation between the 2 variables. LASSO regression was used to eliminate multicollinearity. Statistical analysis was performed using Python 3.7.4 (Anaconda 4.9.2). The main Python library and version information used for modeling are listed in Table 1.

**Figure 1:**
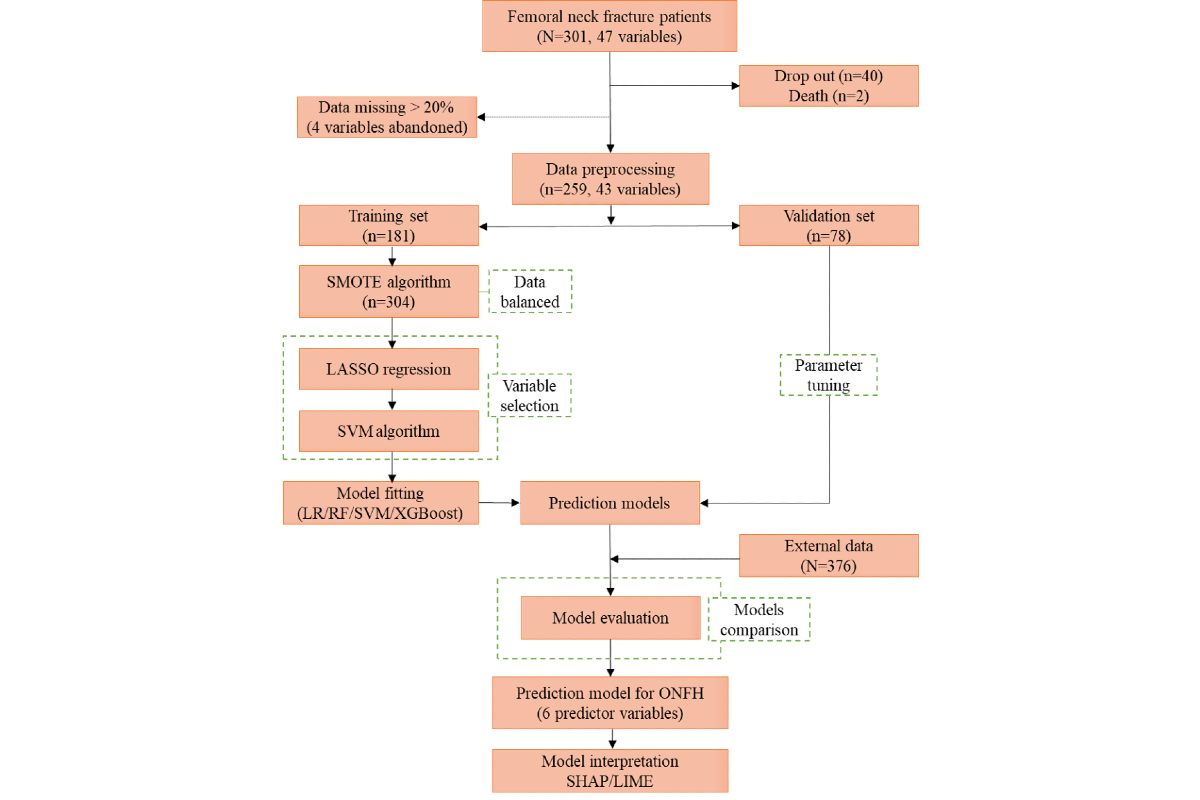
Flowchart of the study. LASSO: least absolute shrinkage and selection operator; LIME: local interpretable model-agnostic explanations; LR: logistic regression; ONFH: osteonecrosis of the femoral head; RF: random forest; SHAP: Shapley additive explanations; SMOTE: synthetic minority oversampling technique; SVM: support vector machine; XGBoost: eXtreme Gradient Boosting.

The flowchart of the study is shown in Figure 1.

**Table 1:** Python library and function.

Library	Version	Function
scikit-learn	0.24.1	Machine learning
NumPy	1.16.5	Scientific computing
pandas	0.25.1	Data analysis
Matplotlib	3.3.4	Visualization
imblearn	0.0	Imbalanced data set
statsmodels	0.12.2	Statistical computations
XGBoost^a^	1.3.3	Gradient boosting framework
SHAP^b^	0.39.0	Explain the output of machine learning model
LIME^c^	0.2.0.1	Explain the output of machine learning model
Flask	1.1.1	Web development
Gunicorn	20.1.0	HTTP server

^a^XGBoost: eXtreme Gradient Boosting.

^b^SHAP: Shapley additive explanations.

^c^LIME: local interpretable model-agnostic explanations.

## Results

### Patient Characteristics

A total of 259 patients with FNF were included in this study, comprising 124 (47.8%) men and 135 (52.1%) women, and the median (P_25_, P_75_) age was 57 (49, 62) years. A total of 43 patients experienced ONFH after internal fixation surgery, with an incidence of ONFH of 16.6%. All data were randomly divided into a training set (181/259, 69.8%) and a validation set (78/259, 30.1%) at a ratio of 7:3 (randomstate=420). There were 29 patients with ONFH and 152 patients without ONFH in the training set. After using the SMOTE algorithm to oversample the femoral head necrosis group in the training set, the number of ONFH and non-ONFH groups reached a balance (152 cases in each group). There were 14 patients with ONFH and 64 patients without ONFH in the validation set. Patient characteristics in the 3 data sets are presented in Multimedia Appendix 2. Overall, the composition of the patients’ variables in the 3 data sets was the same. The results of the feature correlation analysis are presented in Multimedia Appendix 3.

### Variable Selection

**Figure 2:**
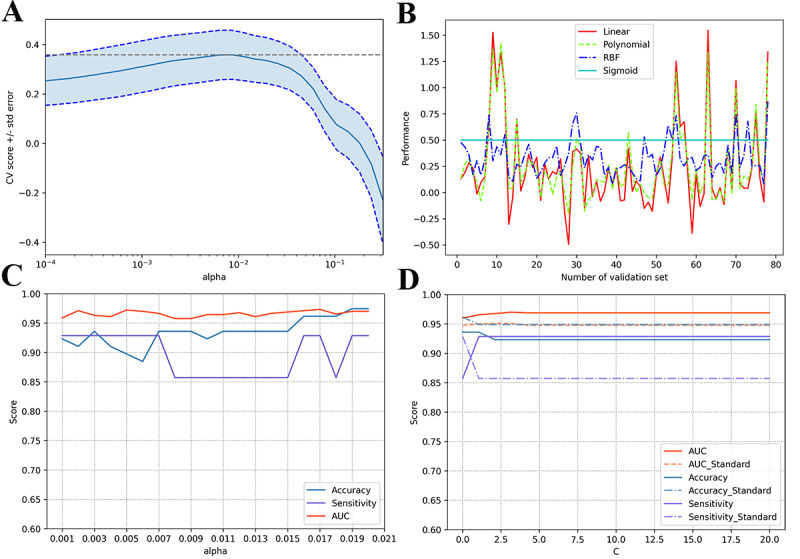
The process of exploring the optimal feature subset. (A) Grid search and 10-fold cross-validation estimators of least absolute shrinkage and selection operator regression regularization parameter α. The y-axis represents the average and SD of 10 cross validations. (B) Prediction results of support vector machine (SVM) with different validation samples under 4 kernel functions of linear, polynomial, radial basis function (RBF) and sigmoid. (C) Best α that makes the SVM model have the best accuracy, sensitivity, and area under the receiver operating characteristic curve performance on the validation set. (D) Comparison of standardized with nonstandardized results of continuous variables under different support vector machine parameters C (kernel=linear). AUC: area under the receiver operating characteristic curve; CV: cross validation.

First, we used the grid search and 10-fold cross-validation estimators (GridSearchCV) to explore the LASSO regression regularization parameter α (Figure 2A). The results of the cross-validation showed that the optimal α was 0.0016. Second, we used the LassoCV object that sets its α parameter automatically from the data by internal cross-validation and used external cross-validation to evaluate the reliability of the selection of α. After 3 cross validations, we obtained 3 alphas for different subsets of the data, which were 0.00646, 0.00281, and 0.00281. However, the scores for these alphas differed substantially, with 0.49697, 0.76142, and 0, respectively. It can be seen that the reliability of LASSO for selecting variables is not very high.

Therefore, to obtain a reliable α and identify the optimal feature subset, the feature subset under the 10-fold cross-validation was first introduced into the SVM classifier for modeling. The performance of the SVM classifier on the validation set using different kernel functions was determined (other parameters used default values). Figure 2B shows the prediction results of SVM with different validation samples under 4 kernel functions: linear, polynomial, radial basis function, and sigmoid. Figure 2B shows that the linear kernel performs best. Next, we identified an α between 0.001 and 0.02, which made the SVM model have the best accuracy, sensitivity, and AUC performance on the validation set. Figure 2C shows that when the α was 0.017, the total accuracy, sensitivity, and AUC were the best. The optimal feature subset included age, sex, time to surgery, injury cause, low energy, fracture position, subcapital, fracture position, neck-to-head, Garden classification Ⅳ, reduction quality, interval to weightbearing, femoral neck shortening, and VAS score.

In addition, we compared the results of standardization with nonstandardization on the accuracy, sensitivity, and AUC of the verification set under different SVM parameters C (kernel=*linear*). In Figure 2D, the solid line is the result of no standardized treatment for continuous variables, and the dotted line is the result of standardization. The figure shows that the performance of the model decreased after the standardization of continuous variables. Therefore, continuous variables were not standardized.

### Modeling and Parameter Tuning

After confirming the optimal feature subset, LR, RF, SVM, and XGBoost algorithms were selected to fit the models on the balanced training set. Table 2 presents the comparison of accuracy, sensitivity, and AUC on the validation set before and after tuning the parameters. The LR and SVM models did not significantly improve. However, the accuracy, sensitivity, and AUC of the RF model were increased by 0.012, 0.072, and 0.006, respectively, and those of the XGBoost model were increased by 0.025, 0.072, and 0.003, respectively. The hyperparameters tuned in each of the 4 classifiers are listed in Table 3.

**Table 2:** Comparison of model performance on the validation set.

Model	Before tuning				After tuning		
	Accuracy	Sensitivity	AUC^a^	Accuracy		Sensitivity	AUC
LR^b^	0.962	0.929	0.982	0.962		0.929	0.984
RF^c^	0.962	0.857	0.985	0.974		0.929	0.991
SVM^d^	0.962	0.929	0.973	0.962		0.929	0.979
XGBoost^e^	0.962	0.857	0.989	0.987		0.929	0.992

^a^AUC: area under the receiver operating characteristic curve.

^b^LR: logistic regression.

^c^RF: random forest.

^d^SVM: support vector machine.

^e^XGBoost: eXtreme Gradient Boosting.

**Table 3:** Hyperparameter configuration for algorithms.

Algorithm and parameter name		Initial value	Adjustment range	Result
**LR^a^**				
	Penalty	L1	(L1, L2)	L2
	C	0.3	(0.1, 0.2, 0.3, 0.4, 0.5, 0.6, 1)	0.5
**RF^b^**				
	n_estimators	100	Range (0, 200, 10)	11
	max_depth	8	(1, 3, 5, 6, 7, 8, 9, 10, 15, 20)	8
	max_features	3	(2, 3, 4, 5, 6, 7, 8)	5
	min_samples_leaf	1	(1, 2, 3, 4)	2
**SVM^c^**				
	Kernel	Rbf	(Linear, polynomial, rbf, sigmoid)	Linear
	C	1	Range (0.01, 20, 20)	7.37
**XGBoost^d^**				
	n_estimators	100	Range (0, 200, 10)	51
	max_depth	6	Range (1, 20, 1)	8
	min_child_weight	1	(2, 3, 4, 5, 6, 7, 8, 9, 10, 15, 20)	7
	learning_rate	0.3	(0.3, 0.31, 0.32, 0.33, 0.335, 0.36, 0.38, 0.4)	0.335
	gamma	0	(0, 1, 2, 3, 4)	1

^a^LR: logistic regression.

^b^RF: random forest.

^c^SVM: support vector machine.

^d^XGBoost: eXtreme Gradient Boosting.

**Figure 3:**
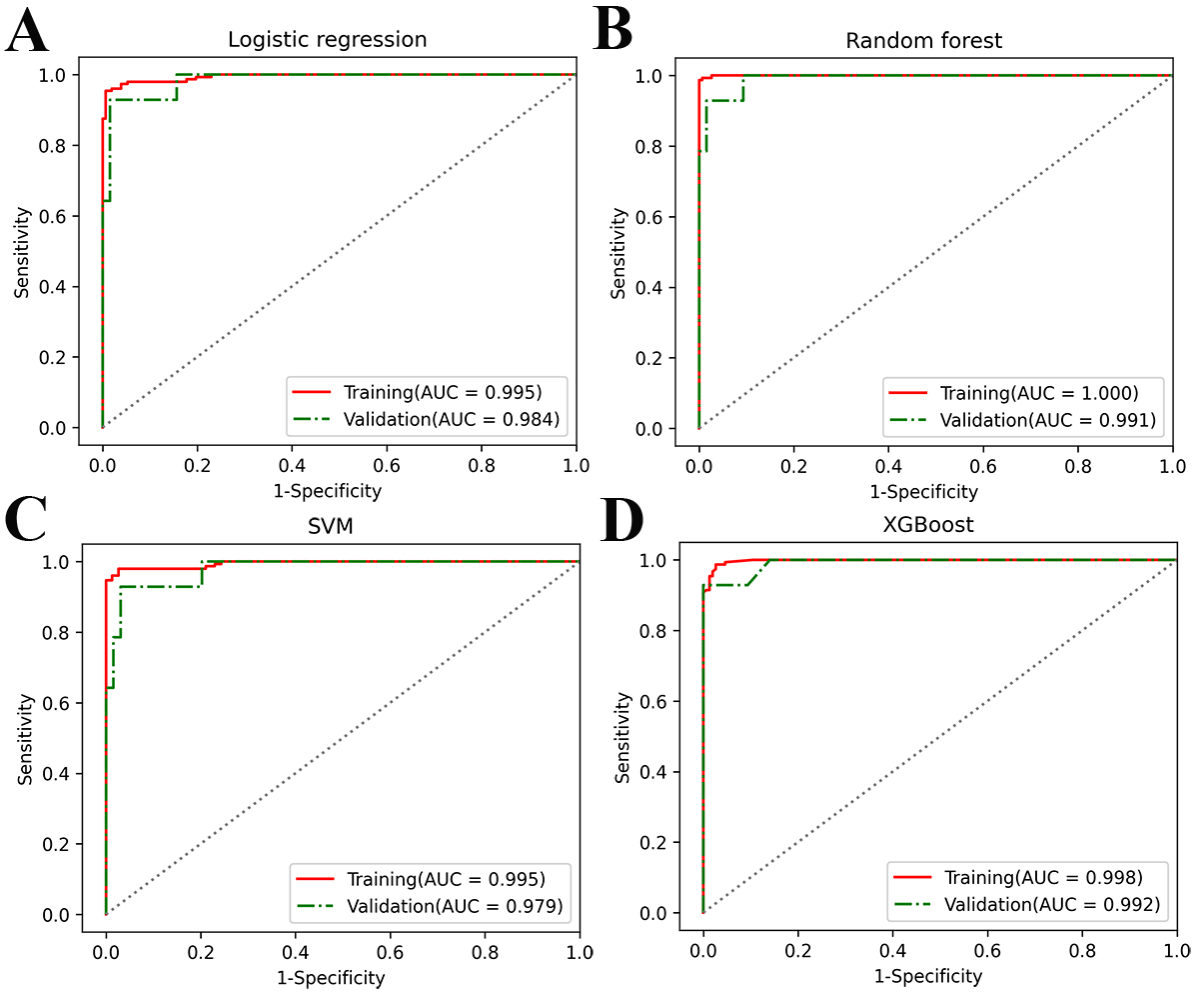
Receiver operating characteristic curves of the logistic regression, random forest, support vector machine (SVM) and eXtreme Gradient Boosting (XGBoost) prediction models on the training set and the validation set, which indicate discrimination ability. The more convex the upper left corner of the curve, the better. AUC: area under the receiver operating characteristic curve.

**Figure 4:**
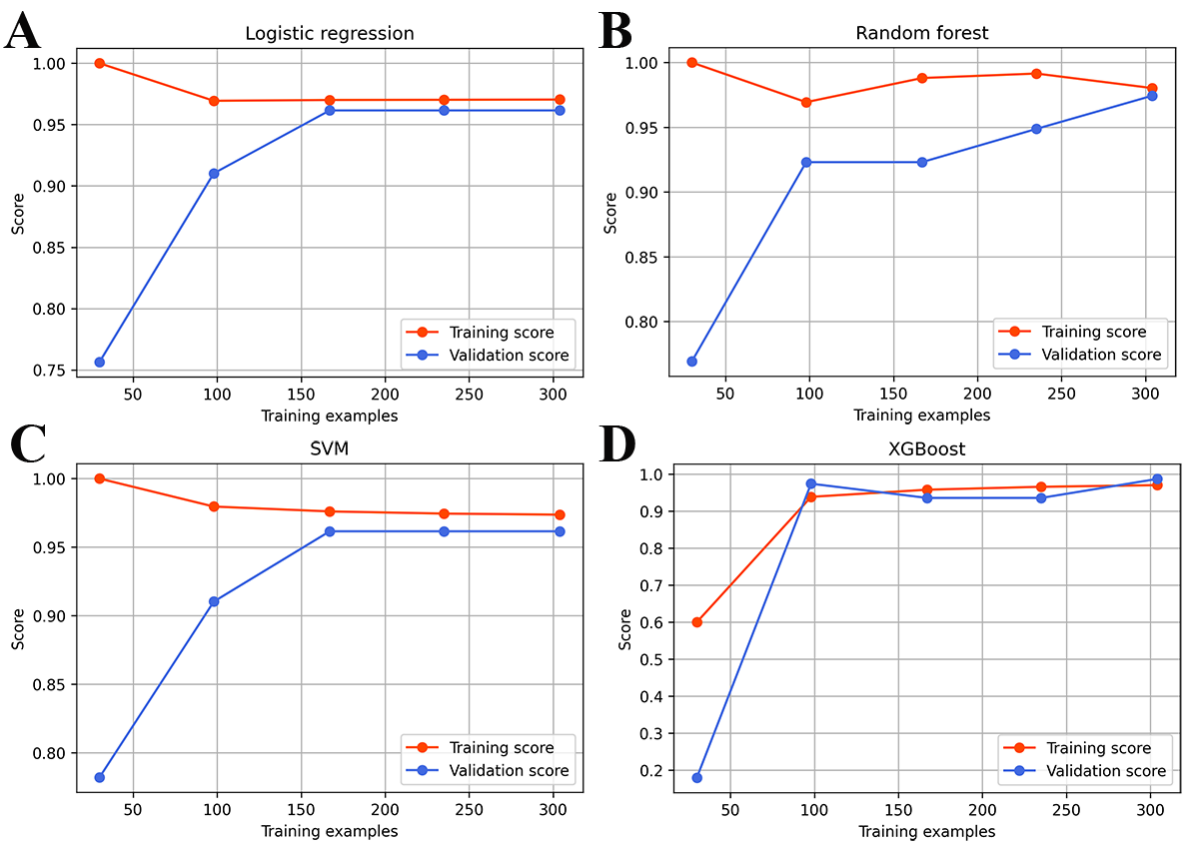
The learning curves of the logistic regression, random forest, support vector machine (SVM) and eXtreme Gradient Boosting (XGBoost) prediction models on the training and validation sets. The 2 curves of logistic regression, support vector machine and XGBoost model are consistent at a higher accuracy level, which indicates that the model is well fitted for training. The 2 curves of random forest are not well merged, indicating that they are slightly overfitted.

Figure 3 shows the ROC curves of the 4 models for the training and validation sets. The AUC values of each algorithm were similar, and the XGBoost model had the highest AUC value in the validation set. Figure 4 shows the learning processes of the 4 models. Except for the RF model, which exhibited slight overfitting when the number of training samples was less than 250, all other models fit well. The parameter configuration of the machine learning models is shown in Multimedia Appendix 4.

### Model Evaluation and Comparison

**Figure 5:**
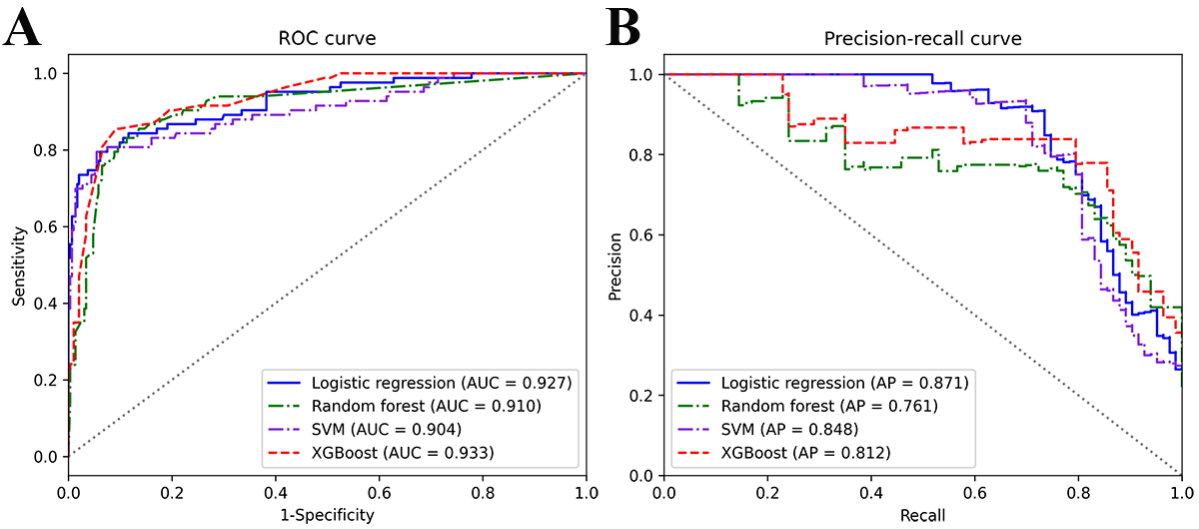
(A) Comparison of receiver operating characteristic curves of the 4 models on external data. The curve closer to the upper left corner showed better overall discrimination ability. (B) Comparison of precision-recall curves of the 4 models on external data. The curve closer to the upper right corner also showed the ability to combine precision with sensitivity. AP: average precision; AUC: area under the receiver operating characteristic curve; ROC: receiver operating characteristic; SVM: support vector machine; XGBoost: eXtreme Gradient Boosting.

**Figure 6:**
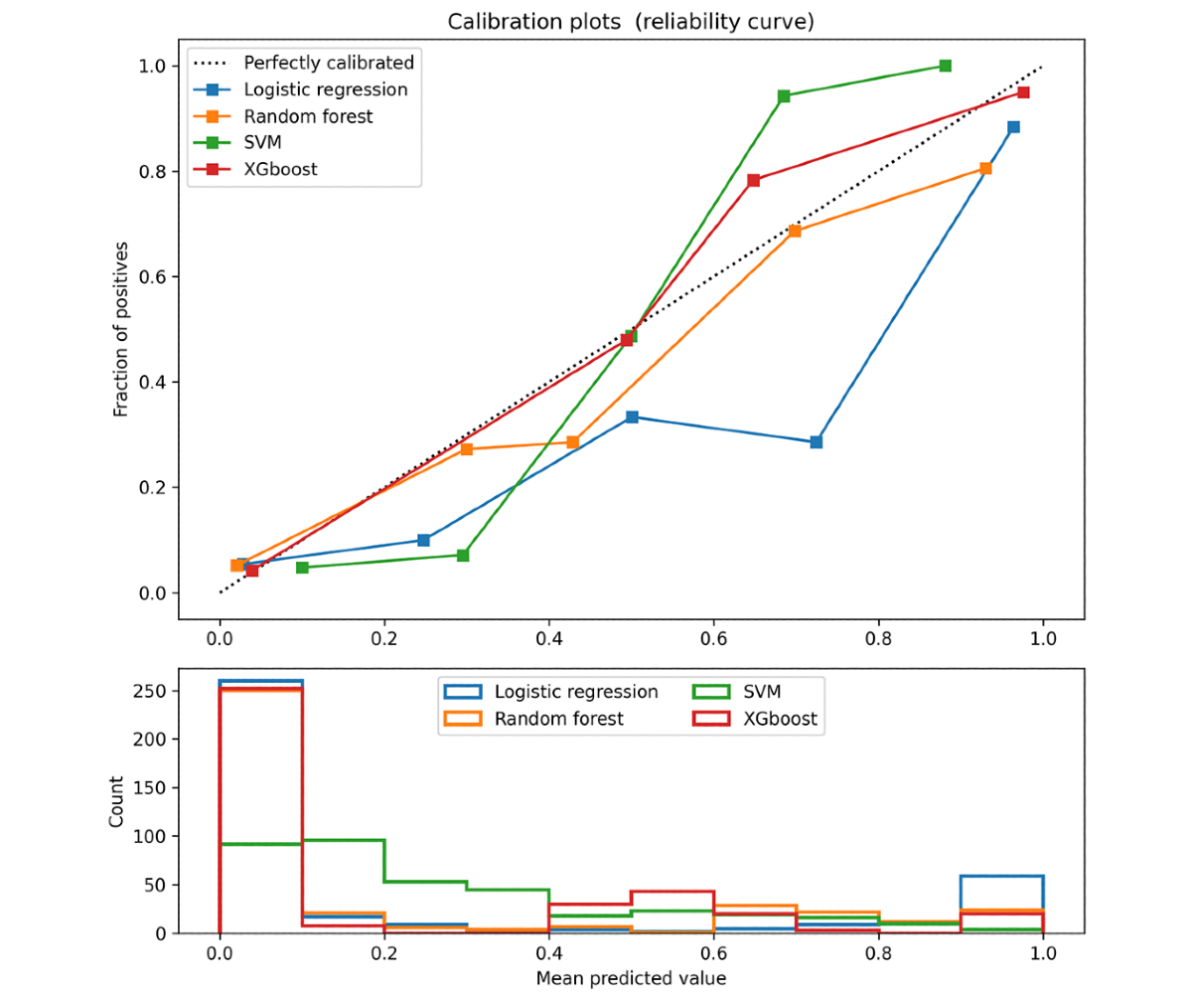
Comparison of calibration curves of the 4 models on external data. The calibration curve of the model is consistent with the ideal calibrated line (y=x), indicating that the predicted value of the model is close to the actual probability of the outcome. SVM: support vector machine; XGBoost: eXtreme Gradient Boosting.

Table 4 presents the confusion matrix of the classification results for the 4 models on the external data. XGBoost generated the maximum number of ONFH (67), and SVM generated the maximum number of non-OFNH (278). Table 5 presents the evaluation results of the 4 models for the external validation data. The XGBoost model exhibited the highest accuracy (0.907). With the exception of the specificity of the XGBoost model being lower than SVM (0.949), the sensitivity (0.807), AUC (0.933), and F1 score (0.793) were all higher than those of the other models. In addition, the XGBoost model presented the smallest log-loss (0.279). A comparison of the ROC curves of the 4 models is shown in Figure 5A. The AUCs of the 4 models were above 0.9, and the XGBoost model achieved the largest AUC. The shapes of the 4 ROC curves were similar. A comparison of the PR curves of the 4 models is shown in Figure 5B. When the sensitivity of the prediction model was greater than 0.7, the XGBoost model had the highest prediction precision. In addition, the LR model achieved the largest AP. The calibration curve is shown in Figure 6, and the curve of the XGBoost model was closest to the ideal calibrated line (y=x).

**Table 4:** Confusion matrices of the prediction models of ONFH^a^.

Model and actual		Predictive	
		ONFH	Non-ONFH
**LR^b^**			
	ONFH	64	19
	Non-ONFH	21	272
**RF^c^**			
	ONFH	64	19
	Non-ONFH	24	269
**SVM^d^**			
	ONFH	61	22
	Non-ONFH	15	278
**XGBoost^e^**			
	ONFH	67	16
	Non-ONFH	19	274

^a^ONFH: osteonecrosis of the femoral head.

^b^LR: logistic regression.

^c^RF: random forest.

^d^SVM: support vector machine.

^e^XGBoost: eXtreme Gradient Boosting.

**Table 5:** Performance comparison on external data.

Model	Accuracy	Discrimination					Calibration
		Sensitivity	Specificity	AUC^a^	F1 score	Log-loss	
LR^b^	0.894	0.771	0.928	0.927	0.762	0.288	
RF^c^	0.886	0.771	0.918	0.910	0.749	0.775	
SVM^d^	0.901	0.735	0.949	0.904	0.767	0.327	
XGBoost^e^	0.907	0.807	0.935	0.933	0.793	0.279	

^a^AUC: area under the receiver operating characteristic curve.

^b^LR: logistic regression.

^c^RF: random forest.

^d^SVM: support vector machine.

^e^XGBoost: eXtreme Gradient Boosting.

### Interpretability of the Prediction Model

**Figure 7:**
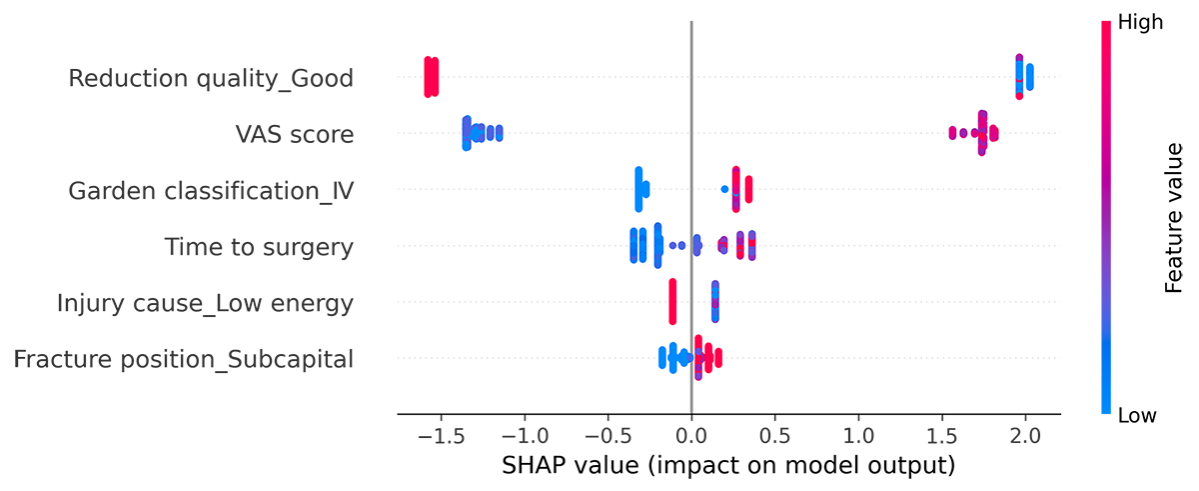
Global explanations of the eXtreme Gradient Boosting model based on Shapley additive explanations (SHAP) values. Summary of the SHAP values of each feature in each sample. The abscissa is the SHAP value (the impact on the model output), the ordinate is the different features, a point represents a sample, and the color represents the feature value. The larger the feature value is, the redder the color, and the smaller the feature is, the bluer the color. VAS: visual analog scale.

On the basis of the above comparisons, we determined that the XGBoost model was the best predictive model for ONFH. Take the average of the absolute value of the SHAP of each feature as the importance of the feature. The predictor variables of the XGBoost model and their importance ranking are as follows: reduction quality (1.759), VAS score (1.483), Garden classification (0.299), time to surgery (0.247), cause of injury (0.127), and fracture position (0.090). Figure 7 shows a summary of the SHAP values of each feature in each sample. The color represents the feature value, and the redder the color, the greater the feature value. Therefore, we can see that the VAS score, Garden classification IV, time to surgery, and fracture position_subcapital are all risk factors for ONFH. Reduction quality_good and injury cause_low energy are protective factors for femoral head necrosis.

**Figure 8:**
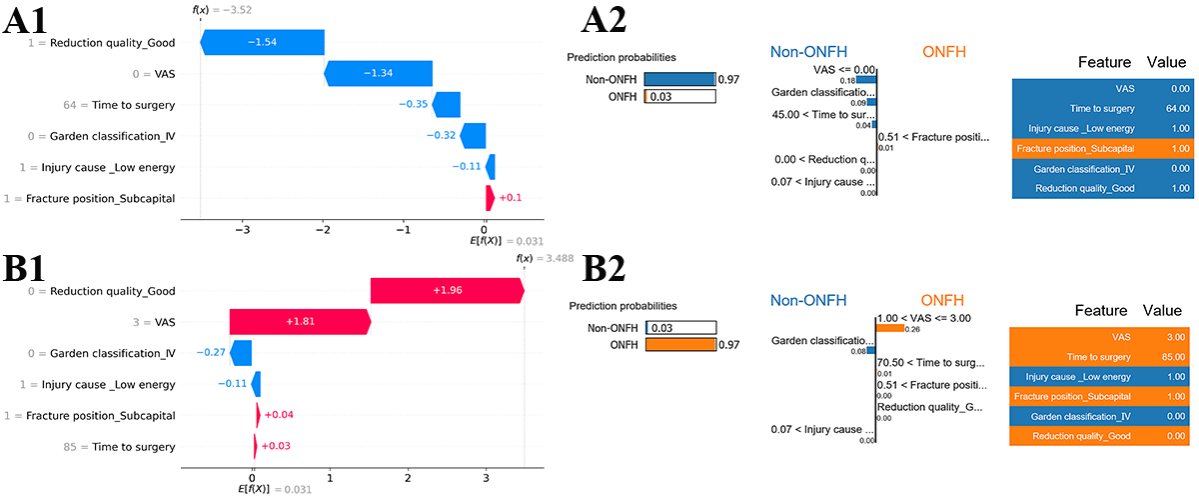
Local explanations of the XGBoost model. (A) The true outcome is nonosteonecrosis of the femoral head (ONFH), and the predicted outcome is non-ONFH. (B) The true outcome is ONFH, and the predicted outcome is ONFH. A1 and B1 are local explanations realized by Shapley additive explanations. Variables in blue decided the sample to be classified into category non-ONFH, and variables in red decided the sample to be classified into category ONFH. A2 and B2 are local explanations realized by local interpretable model-agnostic explanation (LIME). LIME can obtain the probability value of each category, and showing which variables determine the sample to be classified into category non-ONFH (blue) and which variables determine the samples to be classified into category ONFH (orange), specifically listing the numerical size of the samples in these features. VAS: visual analog scale.

Figure 8 shows the decision process for the single-sample prediction. These are local explanations of XGBoost model based on SHAP and LIME. The true outcome of the first sample is non-ONFH, and the predicted outcome is non-ONFH, as shown in Figure 8A. The true outcome of the second sample is ONFH, and the predicted outcome is ONFH, as shown in Figure 8B. The 2 figures on the left and the 2 figures on the right are the results of local explanations by the SHAP and LIME algorithms, respectively. As can be seen from A1 and A2, both SHAP and LIME show that the features determined the outcome of non-ONFH, including reduction quality_good (1), VAS score (0), time to surgery (64), Garden classification_IV (0), and injury cause_Low energy (1). The difference is that LIME can provide a predicted probability of non-ONFH of 0.97. Similarly, B1 and B2 show that the features determined the outcome of ONFH, including reduction quality_good (0), VAS score (3), fracture position_subcapital (1), and time to surgery (85). LIME also shows that the prediction probability of the ONFH is 0.97.

## Discussion

### Principal Findings

In this study, we compared the application of different machine learning algorithms in the prediction of femoral head necrosis after internal fixation of FNFs and obtained a 6-variable XGBoost model that could be used for the clinical prediction of traumatic ONFH. This model was translated into a self-made web-based risk calculator to estimate an individual’s probability of ONFH. The predictors included reduction quality, VAS score, Garden classification, time to surgery, cause of injury, and fracture position. This prediction model exhibited good discrimination and calibration and showed good generalization performance on external data. Performance on the internal validation set yielded an accuracy of 0.987, sensitivity of 0.929, and AUC of 0.992. Performance on external data revealed an accuracy of 0.907, sensitivity of 0.807, specificity of 0.935, AUC of 0.933, F1 score of 0.793, and log-loss of 0.279. The web-based risk calculator can be found on the Herokuapp website [46].

While constructing the predictive model, we also conducted an excavation on the predictive variables of ONFH after internal surgery for FNF. In the design stage of the study, we made our best effort to collect relevant injury and clinical information throughout the clinical course, such as preoperative coagulation indicators, preoperative routine blood tests, and other indicators that have not been analyzed in previous studies. However, these indicators did not pass variable selection. A new British study [47] revealed that poor nutritional status was correlated with mortality and worse postoperative outcomes in patients with FNF. We did not find any indices related to femoral head necrosis on preoperative biochemical examination. Huang et al [48] reported that compared with open reduction in pursuit of anatomical reduction, which may cause vascular injury, positive support can provide reasonable reduction support and reduce the occurrence of vascular necrosis. When Gotfried reduction had a positive buttress pattern, the medial cortex of the distal end of the fracture straddled the *medial femoral neck support bridge* due to sliding compression of the femoral head. The special stress transfer effect of the arch structure can effectively resist the longitudinal shear force between the fracture pieces and stabilize the fracture. We used Gotfried reduction as a predictive variable to participate in the model. However, the overall performance of the model decreased. This is similar to the results reported by Zhao et al [49]. Other controversial risk factors that might be related to femoral head necrosis, such as early weight bearing, removal of internal fixation implants, and reduction methods, were not selected by the classification model.

Among the 6 predictors in the XGBoost model, poor reduction, severe fracture displacement, and delay in operation time were clear risk factors for ONFH after internal fixation of FNF. The VAS pain score is widely used in clinical prognosis research and has high reliability and validity. After internal fixation, patients generally experience slight soreness when they get up and sit down and when the temperature suddenly drops. When osteocytes of the hip joint change histologically, patients may experience pain. Through finite element analysis based on biomechanics, Li et al [50] reported that when necrosis occurs, the increase in mechanical load on the hip joint in patient’s daily life will increase the area of necrotic lesions, especially lesions in the anterior and lateral areas of the femoral head, which are more likely to accelerate expansion and collapse in advance. The causes and mechanisms of postoperative hip pain have not been fully explored and require further investigation. A subcapital fracture indicates that the fracture line is completely located at the bottom of the femoral head. When a subcapital fracture of the femoral neck occurs, it is usually accompanied by a rupture of the medial and lateral femoral circumflex artery, in which the epiphyseal artery from the medial femoral circumflex artery supplies most of the blood to the femoral head. Subcapital fractures are usually accompanied by rupture of the medial and lateral femoral circumflex arteries. However, the epiphyseal artery from the medial circumflex femoral artery supplies most of the blood to the femoral head. The lateral epiphyseal artery is the primary blood vessel for the femoral head. Injury causes most of the blood supply to the femoral head to be interrupted. Only the internal artery of the round ligament supplies the blood. Its blood vessels are thin and supply the femoral head over a small range, but it cannot provide the necessary blood volume to the entire femoral head. Therefore, the necrosis rate of subcapital fractures is higher [51]. Different trauma mechanisms cause different fracture injuries. In FNFs caused by high-energy traumas, such as road traffic accidents and falling from a height, the displacement of the broken end is usually large, often tearing the ascending cervical branches that stem off the arterial ring supply formed by the circumflex arteries, destroying the blood supply to the femoral head and causing nonunion of the fracture or complications, such as femoral head necrosis [52]. The cause of injury has also been considered a risk factor for femoral head necrosis [53].

It is worth noting that before the categorical variables entered the machine learning classifier to fit the model, they were converted into dummy variables according to the category. The Garden classification is a 4-category variable. After XGBoost modeling, only Garden classification IV became a predictor variable. At this time, Garden classification is no longer a 4-category variable but becomes a 2-category variable of Garden classification_IV.

Obtaining a sufficient number of training samples is difficult and time-consuming for the prediction of femoral head necrosis after FNF. LR, RF, SVM, and XGBoost can learn effectively from a limited training set. As a strongly integrated algorithm, the performance of XGBoost was not only better than that of SVM and RF but also more accurate and reliable than traditional LR in our study.

In addition, we opened the black box of machine learning with the help of post hoc interpretability techniques of the machine learning model. Through the global interpretation based on SHAP, we can understand the relationship between predictors and outcomes in the XGBoot model. The variables of reduction quality_good and injury cause_low energy correlated negatively with the outcomes and were protective factors; VAS score, Garden classification_IV, time to surgery, and fracture position_subcapital correlated positively with the outcomes and were risk factors. Both SHAP and LIME can provide local explanations for a single sample. The explanatory plot produced by the SHAP is close to the one generated by LIME in that it shows the variables’ names and contributions that are used in the explanation [54]. The advantage of the LIME method is that the explanation is based on the local regression model, which allows physicians to make statements about changes in explanations for changes in the features of the patient to be explained. The disadvantage is that the instability of the explanations is insufficient. For a single sample, if you get the explanation twice, you may have 2 different explanations. Comparably, the principle of the SHAP method is strictly improved from the classical Shapley value estimation method [55], so the interpretation results of the SHAP algorithm have variable consistency and model stability.

### Limitations

This study has several limitations. First, the number of ONFH cases was insufficient. According to the requirements of PROBAST for the number of participants in clinical events, the ratio of participants in clinical events to the number of candidate predictors was at least 10. There were 6 predictors in the model, and only 43 patients had ONFH. However, we used the SMOTE algorithm to balance the training set and increase the number of ONFH to 152 cases. Second, the sensitivity and F1 score on the external data were approximately 0.8, which is low compared with other indicators. When using the LIME algorithm to explain individual predictions, we discovered that most samples used only 4 variables for prediction. Therefore, the reasons for the low sensitivity and F1 score may include the following: (1) the number of ONFH is insufficient and (2) there are still risk factors related to ONFH that have not been identified. In the future, we will conduct prospective validation based on this model, continue to explore important risk factors for ONFH, and modify the model to further improve the accuracy of the XGBoost prediction model.

### Comparison With Prior Work

The patients with FNF in this study were from 6 hospitals in Shanghai, which are more representative. We included a wider range of candidate variables. Instead of using traditional single-variable analysis for variable selection, LASSO was integrated into the SVM as a new variable selection method. The performance of our model on the validation set was better than that of the naive Bayesian prediction model proposed by Cui et al [24], whose accuracy, sensitivity, and AUC were 0.744, 0.742, and 0.746, respectively. The AUC of our model on the validation set was higher than that of the hybrid nomogram based on LR developed by Zhu et al (0.948) [26] and the nomogram based on Cox regression developed by Zheng et al (0.97) [25]. It also exhibited satisfactory generalization ability on external data, with accuracy, specificity, AUC, and log-loss values of 0.907, 0.935, 0.933, and 0.279, respectively.

### Conclusions

Machine learning performs well in predicting ONFH after internal fixation of FNF. The 6-variable XGBoost model predicts the risk of ONFH well and has good generalization ability in external data, which can be used for the clinical prediction of ONFH after internal fixation of FNF.

## Appendix

Multimedia Appendix 1 Variables and definitions.

Multimedia Appendix 2 Characteristics of 3 groups of patients with femoral neck fracture.

Multimedia Appendix 3 Correlation coefficient matrix heat maps.

Multimedia Appendix 4 Parameters of machine learning models.

## Abbreviations

AP: average precision
AUC: area under the receiver operating characteristic curve
FNF: femoral neck fracture
FPR: false-positive rate
LASSO: least absolute shrinkage and selection operator
LIME: local interpretable model-agnostic explanation
LR: logistic regression
ONFH: osteonecrosis of the femoral head
PR: precision-recall
PROBAST: Prediction Model Risk of Bias Assessment Tool
RF: random forest
ROC: receiver operating characteristic
SHAP: Shapley additive explanations
SMOTE: synthetic minority oversampling technique
SVM: support vector machine
VAS: visual analog scale
XGBoost: eXtreme Gradient Boosting
