# Supplementary material for: Prediction Model of Osteonecrosis of the Femoral Head After Femoral Neck Fracture: Machine Learning–Based Development and Validation Study
Source: JMIR Med Inform. 2021 Nov 19;9(11):e30079. doi: 10.2196/30079 (PMC8663504; doi:10.2196/30079)
Supplement: Multimedia Appendix 2 [file medinform_v9i11e30079_app2.docx]

## Appendix 2. Characteristics of three groups of patients with femoral neck fracture

| Variables | | All Patients (n=259) | Balanced Training set (n=304) | Validation set (n=78) |
| --- | --- | --- | --- | --- |
| Age, median (*P*_25_, *P*_75_) | | 57(49,62) | 57(51,60) | 56(50,61) |
| **Gender, n (%)** | |  |  |  |
|  | Male | 124(47.9) | 143(47.0) | 40(51.3) |
|  | Female | 135(52.1) | 161(53.0) | 38(48.7) |
| **Injury cause, n (%)** | |  |  |  |
|  | Low energy | 179(69.1) | 189(62.2) | 55(70.5) |
|  | High energy | 80(30.9) | 115(37.8) | 23(29.5) |
| **Injured side, n (%)** | |  |  |  |
|  | Left | 138(53.3) | 182(59.9) | 42(53.8) |
|  | Right | 121(46.7) | 122(40.1) | 36(46.2) |
| Smoking, n (%) | | 6(2.3) | 4(1.3) | 2(2.6) |
| Drinking, n (%) | | 6(1.2) | 1(0.3) | 2(2.6) |
| VAS score | | 1(0,1) | 1.63(1,2) | 1(0,1) |
| ACCI, median (*P*_25_, *P*_75_) | | 1(1,2) | 1(1,3) | 1(1,2.25) |
| **Fracture position, n (%)** | |  |  |  |
|  | Subcapital | 134(51.7) | 154(50.7) | 47(60.3) |
|  | Neck-to-head | 51(19.7) | 69(22.7) | 13(16.7) |
|  | Mid-neck | 63(24.3) | 57(18.8) | 16(20.5) |
|  | Basal | 11(4.2) | 24(7.9) | 2(2.6) |
| **Impaction, n (%)** | |  |  |  |
|  | No | 42(16.2) | 35(11.5) | 10(12.8) |
|  | Yes | 217(83.8) | 269(88.5) | 68(87.2) |
| **VN angle, n (%)** | |  |  |  |
|  | Ⅰ | 65(25.1) | 56(18.4) | 20(25.6) |
|  | Ⅱ | 82(31.7) | 77 (25.3) | 27(34.6) |
|  | Ⅲ | 38(14.7) | 61(20.1) | 7(9.0) |
|  | Ⅳ | 74(28.6) | 110(36.2) | 24(30.8) |
| **Garden classification, n (%)** | | |  |  |
|  | Ⅰ | 64(24.7) | 48(15.8) | 16(20.5) |
|  | Ⅱ | 36(13.9) | 28(9.2) | 11(14.1) |
|  | Ⅲ | 72(27.8) | 93(30.6) | 23(29.5) |
|  | Ⅳ | 87(33.6) | 135(44.4) | 28(35.9) |
| **Femoral neck shortening, n (%)** | | |  |  |
|  | No | 95(36.7) | 75(24.7) | 23(29.5) |
|  | Yes | 164(63.3) | 229(75.3) | 55(70.5) |
| **Preoperative displace, n (%)** | | |  |  |
|  | Non | 101(39.0) | 77(25.3) | 27(34.6) |
|  | Displace | 39(15.1) | 44(14.5) | 13(16.7) |
|  | Displace and rotation | 119(45.9) | 183(60.2) | 38(48.7) |
| Time to surgery (h), median (*P*_25_, *P*_75_) | | 69(44,112) | 70(45,110) | 74(48,119) |
| ASA grade, median (*P*_25_, *P*_75_) | | 2(1,2) | 2(1,2) | 2(1,2) |
| **Type of anesthesia, n (%)** | | |  |  |
|  | Local | 46(17.8) | 41(13.5) | 14(17.9) |
|  | General | 213(82.2) | 263(86.5) | 64(82.1) |
| **Operation method, n (%)** | | |  |  |
|  | Three cannulated screw | 229(88.4) | 266(87.5) | 69(88.5) |
|  | Four cannulated screw | 7(2.7) | 8(2.6) | 1(1.3) |
|  | DHS+ cannulated screw | 3(1.2) | 2(0.7) | 1(1.3) |
|  | Plate+ cannulated screw | 20(7.7) | 28(9.2) | 7(9.0) |
| **Surgical treatment, n (%)** | | |  |  |
|  | Close | 248(95.8) | 293(96.4) | 75(96.2) |
|  | Open | 11(4.2) | 11(3.6) | 3(3.8) |
| **Reduction quality, n (%)** | |  |  |  |
|  | Good | 228(88.0) | 186(61.2) | 69(88.5) |
|  | Poor | 31(12.0) | 118(38.8) | 9(11.5) |
| **Lowell curve, n (%)** | |  |  |  |
|  | No | 79(30.5) | 134(44.1) | 24(30.8) |
|  | Yes | 180(69.5) | 170(55.9) | 54(69.2) |
| **Gotfried reduction, n (%)** | |  |  |  |
|  | Anatomic reduction | 184(71.0) | 177(58.2) | 56(71.8) |
|  | Negative | 39(15.1) | 79(26.0) | 12(15.4) |
|  | Positive | 36(13.9) | 48(15.8) | 10(12.8) |
| **PT, n (%)** | |  |  |  |
|  | Low | 156(60.2) | 174(57.2) | 42(53.8) |
|  | Normal | 93(35.9) | 116(38.2) | 35(44.9) |
|  | High | 10(3.9) | 14(4.6) | 1(1.3) |
| **FIB, n (%)** | |  |  |  |
|  | Low | 17(6.6) | 16(5.3) | 6(7.7) |
|  | Normal | 212(81.9) | 253(83.2) | 66(84.6) |
|  | High | 30(11.6) | 35(11.5) | 6(7.7) |
| **APTT, n (%)** | |  |  |  |
|  | Lower | 27(10.4) | 41(13.5) | 6(7.7) |
|  | Normal | 232(89.6) | 263(86.5) | 72(92.3) |
| **INR, n (%)** | |  |  |  |
|  | Normal | 254(98.1) | 293(96.4) | 78(100) |
|  | High | 5(1.9) | 11(3.6) | 0(0) |
| **WBC, n (%)** | |  |  |  |
|  | Low | 5(1.9) | 5(1.6) | 0(0) |
|  | Normal | 189(73.0) | 237(78.0) | 51(65.4) |
|  | High | 65(25.1) | 62(20.4) | 27(34.6) |
| **RBC, n (%)** | |  |  |  |
|  | Low | 83(32.0) | 92(30.3) | 24(30.8) |
|  | Normal | 170(65.6) | 209(68.8) | 51(65.4) |
|  | High | 6(2.3) | 3(1.0) | 3(3.8) |
| **Hb, n (%)** | |  |  |  |
|  | Low | 17(6.6) | 26(8.5) | 4(5.1) |
|  | Normal | 238(91.9) | 276(90.8) | 72(92.3) |
|  | High | 4(1.5) | 2(0.7) | 2(2.6) |
| **Platelet, n (%)** | |  |  |  |
|  | Low | 4(2.6) | 7(2.3) | 2(2.6) |
|  | Normal | 147(96.7) | 293(96.4) | 73(93.6) |
|  | High | 1(0.7) | 4(1.3) | 3(3.8) |
| **HCT, n (%)** | |  |  |  |
|  | Low | 107(41.3) | 126(41.4) | 33(42.3) |
|  | Normal | 144(55.6) | 173(56.9) | 42(53.8) |
|  | High | 8(3.1) | 5(1.7) | 3(3.8) |
| **TP, n (%)** | |  |  |  |
|  | Low | 9(3.5) | 16(5.3) | 2(2.6) |
|  | Normal | 239(92.3) | 276(90.8) | 74(94.9) |
|  | High | 11(4.2) | 12(3.9) | 2(2.6) |
| **ALB, n (%)** | |  |  |  |
|  | Low | 10(3.9) | 20(6.6) | 2(2.6) |
|  | Normal | 249(96.1) | 284(93.4) | 76(97.4) |
| **ALB/GLB, n (%)** | |  |  |  |
|  | Low | 142(54.8) | 169(55.6) | 34(43.6) |
|  | Normal | 117(45.2) | 135(44.4) | 44(56.4) |
| **T-bill, n (%)** | |  |  |  |
|  | Normal | 149(57.5) | 191(62.8) | 40(51.3) |
|  | High | 110(42.5) | 113(37.2) | 38(48.7) |
| **ALT, n (%)** | |  |  |  |
|  | Normal | 238(91.9) | 288(94.7) | 72(92.3) |
|  | High | 21(8.1) | 16(5.3) | 6(7.7) |
| **AST, n (%)** | |  |  |  |
|  | Normal | 245(94.6) | 292(96.1) | 73(93.6) |
|  | High | 14(5.4) | 12(3.9) | 5(6.4) |
| **Creatinine, n (%)** | |  |  |  |
|  | Low | 17(6.6) | 26(8.6) | 4(5.1) |
|  | Normal | 238(91.9) | 274(90.1) | 72(92.3) |
|  | High | 4(1.5) | 4(1.3) | 2(2.6) |
| **Uric acid, n (%)** | |  |  |  |
|  | Low | 17(6.6) | 11(3.6) | 6(7.7) |
|  | Normal | 213(82.2) | 256(84.2) | 65(83.3) |
|  | High | 29(11.2) | 37(12.2) | 7(9.0) |
| **Urea nitrogen, n (%)** | |  |  |  |
|  | Low | 2(0.8) | 11(3.6) | 2(2.6) |
|  | Normal | 228(88.0) | 256(84.2) | 66(83.3) |
|  | High | 29 (11.2) | 37(12.2) | 7(9.0) |
| **Implant removal, n (%)** | |  |  |  |
|  | No | 203(78.4) | 253(83.2) | 61(78.2) |
|  | Yes | 56(21.6) | 51(16.8) | 17(21.8) |
| Interval to part weightbearing, median (*P*_25_, *P*_75_) | | 3(2,3) | 3(1.97,3.08) | 3(2,4) |
| Interval to weightbearing, median (*P*_25_, *P*_75_) | | 5(3,7) | 5(4,7) | 6(4,7) |
| **ONFH, n (%)** | |  |  |  |
|  | No | 216(83.4) | 152(50) | 64(82.1) |
|  | Yes | 43(16.6) | 152(50) | 14(17.9) |
