# Supplementary material for: Prediction Model of Osteonecrosis of the Femoral Head After Femoral Neck Fracture: Machine Learning–Based Development and Validation Study
Source: JMIR Med Inform. 2021 Nov 19;9(11):e30079. doi: 10.2196/30079 (PMC8663504; doi:10.2196/30079)

## Multimedia Appendix 3. Correlation coefficient matrix heat maps

Figure A is the heat map of the correlation matrix of continuous variables. The value in the matrix is the Spearman correlation coefficient between two variables. The darker the color in the matrix, the stronger the correlation between variables. Figure B is the heat map of the correlation matrix of categorical variables. The value in the matrix is the Kendall correlation coefficient between two variables. The darker the color in the matrix, the stronger the correlation between variables.


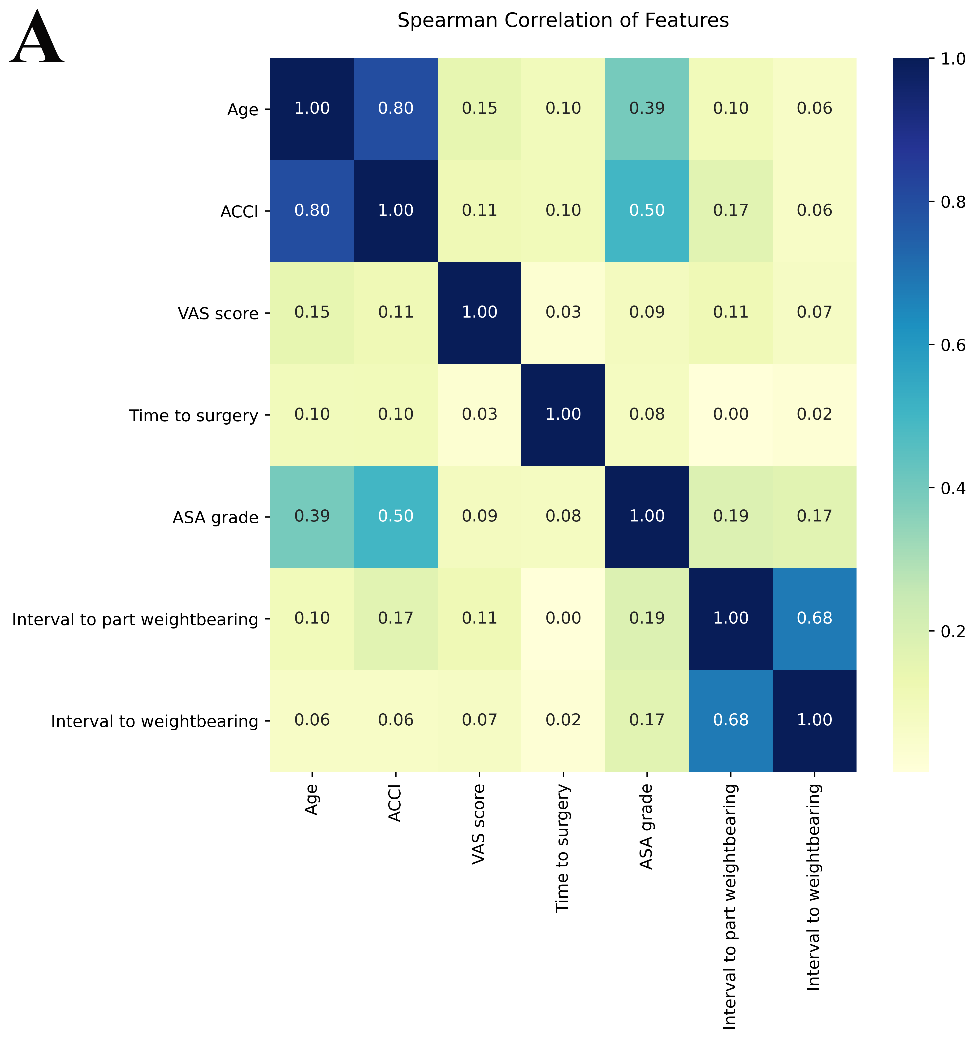


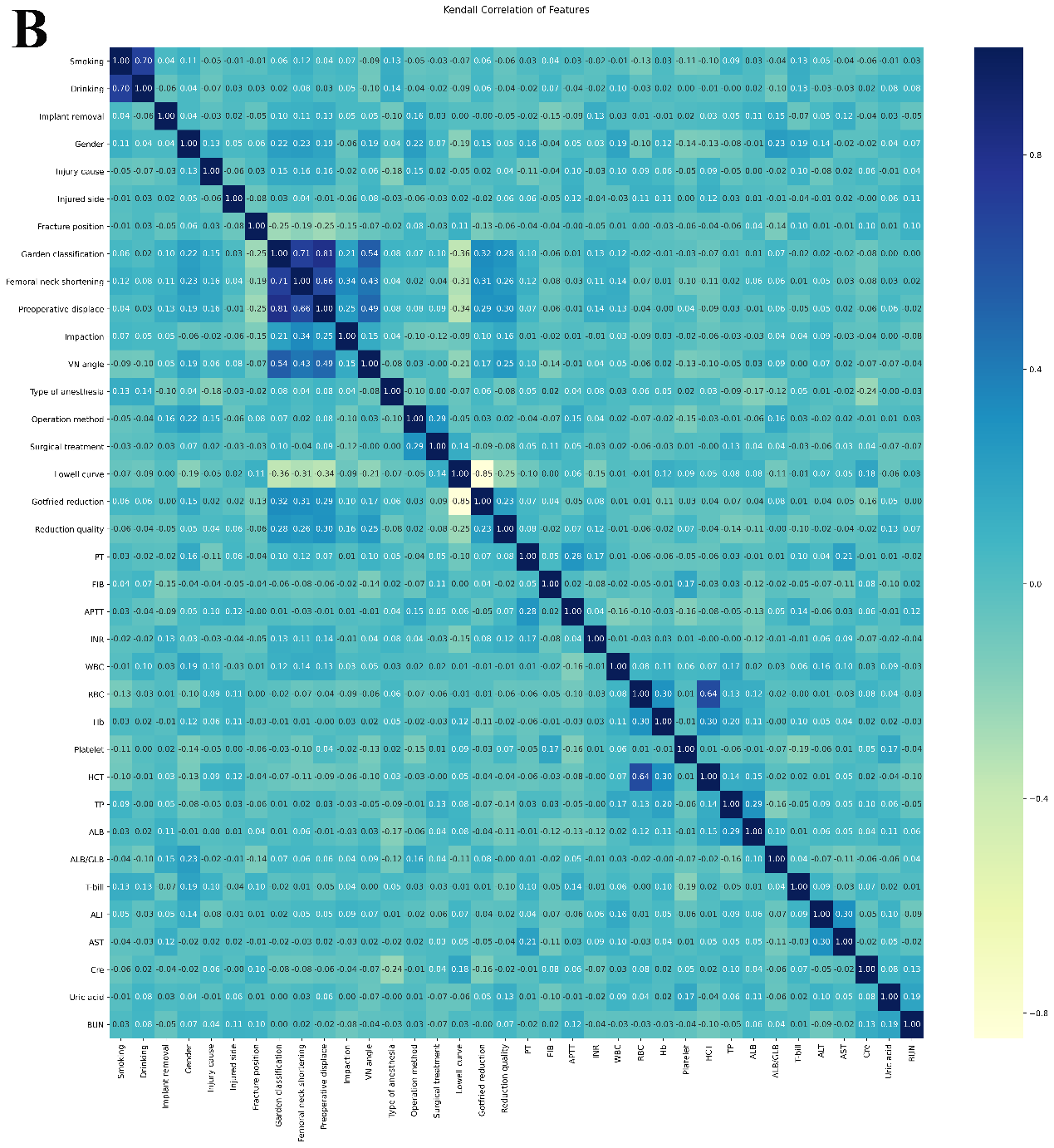

Supplement: Multimedia Appendix 3 [file medinform_v9i11e30079_app3.docx]
