# Supplementary material for: Prediction Model of Osteonecrosis of the Femoral Head After Femoral Neck Fracture: Machine Learning–Based Development and Validation Study
Source: JMIR Med Inform. 2021 Nov 19;9(11):e30079. doi: 10.2196/30079 (PMC8663504; doi:10.2196/30079)
Supplement: Multimedia Appendix 4 [file medinform_v9i11e30079_app4.docx]

## Multimedia Appendix 4. Parameters of machine learning models

| Algorithm | Parameters |
| --- | --- |
| LogisticRegression | LogisticRegression (penalty='l2', C=0.5, class_weight=None, dual=False, random_state=420, solver='liblinear', fit_intercept=True, intercept_scaling=1, l1_ratio=None, max_iter=100, multi_class='warn', n_jobs=None, tol=0.0001, verbose=0, warm_start=False) |
| RandomForest | RandomForestClassifier (n_estimators=11, criterion='gini', max_depth=8, min_samples_split=3, min_samples_leaf=2, max_features=5, min_weight_fraction_leaf=0.0, random_state=420, max_leaf_nodes=None, min_impurity_decrease=0.0, min_impurity_split=None, bootstrap=True, oob_score=False, n_jobs=None, verbose=0, warm_start=False, class_weight=None, ccp_alpha=0.0, max_samples=None) |
| SVM | SVC (C=7.37, kernel='linear', degree=3, gamma='scale', coef0=0.0, random_state=420, shrinking=True, probability=False, tol=0.001, cache_size=200, class_weight=None, verbose=False, max_iter=- 1, decision_function_shape='ovr', break_ties=False) |
| XGBoost | XGBClassifier (n_estimators=51, use_label_encoder=False, max_depth=8, min_child_weight=7, gamma=1, random_state=420, base_score=0.5, booster='gbtree', colsample_bylevel=1, colsample_bynode=1, colsample_bytree=1, learning_rate=0.335, max_delta_step=0, missing=None, n_jobs=1, nthread=None, objective='binary: logistic', reg_alpha=0.1, reg_lambda=1, scale_pos_weight=1, seed=None, silent=None, subsample=1, verbosity=1) |
